# Gender differences in response to medical red packets (Hongbao, monetary gifts): a questionnaire study on young doctors in China

**DOI:** 10.1186/s12910-022-00781-0

**Published:** 2022-04-19

**Authors:** Mengci Yuan, Hanhui Xu

**Affiliations:** School of Medicine, Nankai University, Tianjin, 300071 China

**Keywords:** Medical ethics, Unethical behavior, Gender difference, Red packet

## Abstract

**Background:**

The acceptance of informal payments by doctors is usually viewed as unethical behavior. However, in China, such behavior is a common practice. In this study, we focus on the gender differences in accepting red packets (informal payments) by young doctors in China.

**Methods:**

A total of 413 young doctors were selected for the study, all of whom were grouped by gender. The questionnaire was designed to include general demographic characteristics, whether they had ever been offered red packets, whether they had ever accepted red packets, the reasons for accepting red packets and so on. Wilcoxon rank-sum test, Pearson’s chi-squared test, univariable and multi-variable logistic regressions were used for all analyses by Stata 17.0 SE and *p*-value < 0.05 was considered statistically significant.

**Results:**

Compared to women, men were more likely to be offered red packets (69.5% [180/259] vs.53.9% [83/154]), and the odds ratio (OR) was statistically significant after adjusting for age, education, position and geographical areas (adjusted OR 1.81, *p* = 0.012). In terms of the question of whether or not they had accepted red packets, more male doctors answered “yes” compared to female doctors (33.3% [60/180] vs.15.7% [13/83], adjusted OR 2.80, *p* = 0.004). However, among those who had accepted red packets, we found that only 42.0% [25/60] of male doctors considered that it was normal to accept such red packets, compared to 85.0% [11/13] of women (adjusted OR 12.01, *p* = 0.023).

**Conclusion:**

The study revealed that Chinese patients and their families were more likely to offer red packets to male doctors. Secondly, among doctors who had been offered red packets, male doctors were more likely to accept red packets than female doctors. In addition, among doctors who had accepted red packets, female doctors were more likely to believe that it was not morally wrong to accept such red packets.

**Supplementary Information:**

The online version contains supplementary material available at 10.1186/s12910-022-00781-0.

## Background

A substantial body of research related to gender differences in unethical behavior has indicated that women generally tend to behave more ethically than men. Such research is usually divided into two categories: psychological and behavioral. Psychological studies focus on gender differences in moral emotions, such as being prone to shame and guilt and empathic concern [1–4]. Researchers usually used psychological scales to observe the participants’ emotional reactions. These scales included Guilt and Shame Proneness Scale (GASP), Guilt Inventory (GI), Test of Self-Conscious Affect (TOSCA), and the MoMo Rating Scale. In some cases, participants were asked to select one point varying from 1 to 7 to show their emotions when read prepared scenarios. In such studies, women were often showed more guilt and shame proneness than men. These differences are thought to be important factors that contribute to differences in moral behavior between men and women [4–6].

The empirical studies, on the other hand, focus on differences in the choices made or actions taken by men and women in specific situations. These scenarios often involve a moral dilemma or the possibility of violating ethical codes [7–18]. Previous studies have shown that in business, women are more reluctant than men to become involved in unethical business practices, despite such practices (e.g., fraud in negotiations) leading to monetary gain or social status [8–10, 14, 15]. In academia, studies on academic plagiarism have also shown that female students show more reluctance to plagiarize than male students [11, 16, 18]. This is also confirmed by research in the field of information studies, where women were determined to better identify unethical behavior than men [7]. However, there has been no research on gender differences in unethical behavior in medicine.

Red Packets are informal payments provided by patients and their families to doctors. This form of informal payment is usually defined as "payments to individual and institutional providers in-kind or cash that are outside the official payment channels, or are purchases that are meant to be covered by the healthcare system" [19]. Although the existing literature does not reach a consensus on the definition of informal payment, the mainstream definitions express a common view that it is an additional payment provided by patients to healthcare providers above what they should pay for the healthcare services [19–22]. In China, the acceptance of red packets is a common practice. Patients and family members offer red packets for better services, greater patience and faster access to surgery [23–27]. In China, a patient’s medical bill is either paid by himself or covered by his medical insurances. No matter which payment methods, patients should not do any direct payment to the doctors. Doctors report accepting red packets to compensate for their low income and to reflect confidence on their medical abilities [23–26].

The acceptance of informal payments by doctors is usually viewed as unethical behavior and a form of corruption in healthcare [19, 28–32]. Studies have shown that the acceptance of red packets is one major negative factor affecting the doctor-patient relationship in China [24, 33–35]. The only potential exception of informal payments is so-called “gratitude payments”, which are informal payments offered by patients and their families out of gratitude. Such payments are often provided to doctors by patients after the surgery. It seems that it does not appear to be an ethical problem to accept such informal payments [36]. However, the proportion of gratitude payments is small. In a survey of 4000 inpatients from 10 hospitals in China, 54% respondents said that they had offered red packets to their doctors. Of these, less than 5% claimed that what they offered was for showing gratitude [24]. In addition, in practice, it is difficult to define what kind of informal payments are offered out of gratitude. Post-operative or post-discharge payments do not necessarily mean that they are offered out of gratitude, especially as some patients’ treatments are continuous and recurrent. In order to avoid a slippery slope where doctors accept large bribe payments under the guise of receiving gratitude payments, in China, the acceptance of red packets (regardless of the patients’ motive for offering them) is forbidden by the code of professional ethics for doctors, and the Law on Licensed Doctors of the People's Republic of China (LODC). The aim of this study is therefore to examine whether there are gender differences in accepting red packets among young doctors in China, and the possible reasons behind this.

This research makes at least two contributions to the gender difference research in unethical behavior. Firstly, no previous empirical study has examined gender differences in unethical behavior in the medical field. Secondly, most of the previous empirical studies were scenario-based, which actually revealed the differences in cognitive level for unethical behavior between women and men. Instead, this research is “real world-based”, which is expected to reveal the real reactions and attitudes of doctors to red packets in their everyday working environment. Thus, compared to the former studies, our findings may provide a more realistic picture of the gender differences in unethical behavior.

## Methods

### Survey development

We developed an eleven-item online survey targeted at surgery-related specialties, and the geographical area and province of the participants are not restricted. The survey was administered between December 23, 2020 and January 21, 2021. The survey link was posted via WeChat and the posts were sharable to facilitate snowball sampling. Informed consent was obtained. In this study, the definition of “red packet” has been limited to cash, shopping cards and vouchers given to doctors by patients or their families. Participants were invited to complete an original 11‑item survey focusing on the general demographic characteristics of the subjects (gender, age, education, title, location, etc.), whether they had ever been offered red packets, whether they had accepted red packets, the reasons for accepting red packets if they did so, whether their attitudes towards patients had changed after accepting red packets, and how they viewed their forbidden behavior. In addition, some participants expressed their personal experiences and feelings after completing the questionnaire. Relevant information provided by them was also taken into account in our study.

### Survey participants

Researchers invited Chinese clinical doctors from different departments or institutions to complete a 10-min online survey (Additional file 1) in December 2020, and Snowball sampling was employed to select more participants. The only criterion for inclusion was that participants must be young Chinese doctors with clinical work experience, and participation was voluntary and confidentiality ensured via a consent document (Additional file 2). Completed surveys were stored separately, and were not linked to participants’ names.

### Statistical analysis

We reported the characteristics of participants stratified by gender as a median and interquartile range (IQR), and number and percentage for categorical variables. We used Wilcoxon rank-sum test and Pearson’s chi-squared test to describe the differences between gender for continuous and categorical variables, respectively. The relationship between gender and being offered or accepting red packets were firstly explored using univariable logistic regression models. To control potential confounding, we further adjusted for other socio-demographic factors in the multi-variable logistic regression models. Stata 17.0 SE (College station, StataCorp, TX) was used for all analyses, and a *p*-value < 0.05 was considered statistically significant.

## Results

A total of 413 young doctors participated in the survey, with an average age of 31. Among them, 259 (62.71%) were male and 154 (37.29%) were female; 81 (19.6%) had a doctoral degree, 272 (65.9%) had a master's degree and 60 (14.5%) had a bachelor's degree. In terms of their position, there were 75 (18.2%) interns, 240 (58.1%) residents, 90 (21.8%) attending physicians and 8 (1.9%) chief physicians. The respondents were from different areas in China (Table 1).

**Table 1:** Characteristics of participants by gender (N = 413)

Variables	Total (N = 413)	Female (n = 154)	Male (n = 259)	*p*-value
Age, Median (IQR) years	29 (27–30)	29 (26–30)	30 (28–31)	** < 0.001**
*Education*				0.900
Undergraduate	60 (14.5%)	22 (14.3%)	38 (14.7%)	
Master	272 (65.9%)	100 (64.9%)	172 (66.4%)	
PhD	81 (19.6%)	32 (20.8%)	49 (18.9%)	
*Position*				**0.008**
Intern/trainee	75 (18.2%)	38 (24.7%)	37 (14.3%)	
Resident	240 (58.1%)	89 (57.8%)	151 (58.3%)	
Attending/Chief	98 (23.7%)	27 (17.5%)	71 (27.4%)	
*Geographical areas*				**0.009**
East-North	111 (26.9%)	48 (31.2%)	63 (24.3%)	
Central-South	86 (20.8%)	27 (17.5%)	59 (22.8%)	
East	75 (18.2%)	18 (11.7%)	57 (22.0%)	
West	19 ( 4.6%)	5 ( 3.2%)	14 ( 5.4%)	
North	122 (29.5%)	56 (36.4%)	66 (25.5%)	

Wilcoxon rank-sum test was used for age and for other variables

Bold indicates a *p* < 0.05 (typically < 0.05) is statistically significant

IQR, Interquartile range

Our survey showed that (Table 2), compared to 83 out of 154 (53.9%) female doctors, 180 out of 259 (69.5%) men had been offered a red packet. The univariable logistic regression model suggested that male doctors were more likely to be offered a red packet [unadjusted OR 1.95; 95% Confidence Interval (CI) 1.29, 2.94, *p* = 0.002], and this association was still highly statistically significant after controlling areas for age, education, position and geography (adjusted OR 1.81; 95% CI 1.14, 2.86, *p* = 0.012). Of the participants who answered “had been offered a red packet”, including 83 women and 180 men, we asked if they had accepted such packets, and 13 (15.7%) women and 60 (33.3%) men, 73 in total, said they had accepted the red packet (Table 3, adjusted OR 2.80; 95% CI 1.40, 5.61, *p* = 0.004). Next, we explored the issues associated with the receipt of red packets for these 73 individuals.

**Table 2:** Association between gender and being offered red packets (N = 413)

	n/N	Unadjusted OR	*p*-value	Adjusted OR*	*p*-value
Female	83/154	1.00 (ref)		1.00 (ref)	
Male	180/259	1.95 (1.29, 2.94)	**0.002**	1.81 (1.14, 2.86)	**0.012**

Female as the reference group

Bold indicates a *p* < 0.05 (typically < 0.05) is statistically significant

n/N, the number of participants reported being offered red packet/the number of participants

*Adjusted for age, education, position and geographical areas

**Table 3:** Association between gender and ever accepting red packets (N = 263)

	n/N	Unadjusted OR	*p*-value	Adjusted OR*	*p*-value
Female	13/83	1.00 (ref)		1.00 (ref)	
Male	60/180	2.69 (1.38, 5.25)	**0.004**	2.80 (1.40, 5.61)	**0.004**

Female as the reference group

Bold indicates a *p* < 0.05 (typically < 0.05) is statistically significant

n/N, the number of participants reported being offered red packet/the number of participants

*Adjusted for age, education, position and geographical areas

As shown in Table 4, of the 73, 56 (93.3%) male doctors and 9 (69.2%) female doctors believed that the main factors taken into consideration for accepting the red packets was the patients’ socio-economic level, rather than the complexity of the patients’ disease, the insurance situation, the patients’ education level and so on (*p* = 0.012). As to whether the acceptance of the red packet may change the attitude towards the patient, our results show that only seven 7 (11.7%) male doctors and 1 (7.7%) female doctor stated that they would give priority to the patient, while the majority of doctors claimed that accepting red packets did not affect their attitude. Finally, we wanted to find out how those doctors who had accepted red packets viewed their own behavior. After removing 9 invalid answers(invalid answers include blank answers and the ambiguous answers. These answers seemed to indicate that the participant did not have a clear judgment of the behavior. Thus, we kept the clear attitudes of their behavior and removed meaningless or unclear answers.), the results showed that 25 out of 60 (42.0%) male doctors thought it was normal for Chinese doctors to accept red packets, while 11 out of 13 (85.0%) female doctors thought it was normal (*p* = 0.021). The gender variability in this issue was also significant (Table 5, adjusted OR 12.01; 95% CI 1.41, 102.43, *p* = 0.023).

**Table 4:** Relationship between gender and some other factors that influenced the acceptance of red packages (N = 73)

Variables	Options	Total	Female	Male	*p*-value
		N = 73	N = 13	N = 60	
*The factors of patients taken into consideration*					
Complexity of disease	No	17 (23%)	3 (23%)	14 (23%)	0.98
	Yes	56 (77%)	10 (77%)	46 (77%)	
Socio-economic level	No	8 (11%)	4 (31%)	4 ( 7%)	**0.012**
	Yes	65 (89%)	9 (69%)	56 (93%)	
Insurance situation	No	46 (63%)	7 (54%)	39 (65%)	0.45
	Yes	27 (37%)	6 (46%)	21 (35%)	
Patient education degree	No	49 (67%)	8 (62%)	41 (68%)	0.64
	Yes	24 (33%)	5 (38%)	19 (32%)	
Relationship with the doctors	No	13 (18%)	1 ( 8%)	12 (20%)	0.29
	Yes	60 (82%)	12 (92%)	48 (80%)	
If change attitude after red packet	No	26 (36%)	3 (23%)	23 (38%)	0.30
	Yes	47 (64%)	10 (77%)	37 (62%)	
How to view this behaviors	Normal	36 (49%)	11 (85%)	25 (42%)	**0.021**
	Abnormal	28 (38%)	2 (15%)	26 (43%)	
	Missing	9 (12%)	0 ( 0%)	9 (15%)	

Bold indicates a *p* < 0.05 (typically < 0.05) is statistically significant

**Table 5:** Association between gender and opinions on accepting red packets (N = 73)

Outcome	Unadjusted OR	*p*-value	Adjusted OR*	*p*-value
Complexity of disease	0.99 (0.24, 4.09)	0.984	1.52 (0.27, 8.51)	0.633
Socio-economic level	6.22 (1.31, 29.45)	**0.021**	10.44 (1.43, 76.15)	**0.021**
Insurance situation	0.63 (0.19, 2.11)	0.452	0.23 (0.04, 1.26)	*0.091*
Patient education degree	0.74 (0.21, 2.57)	0.637	0.61 (0.14, 2.55)	0.501
The relationship with the doctors	0.33 (0.04, 2.82)	0.313	0.66 (0.05, 8.27)	0.750
If change attitude after red packet	0.48 (0.12, 1.94)	0.305	0.41 (0.09, 1.89)	0.252
How to view this behaviors- abnormal*	5.72 (1.15, 28.43)	**0.033**	12.01 (1.41, 102.43)	**0.023**

Female as the reference group

Bold indicates a *p* < 0.05 (typically < 0.05) is statistically significant

*Invalid data were excluded (i.e., 9 participants did not respond clearly were excluded)

## Discussion

In China, the acceptance of red packets is forbidden by the code of professional ethics for doctors and LODC. According to the results, there are gender differences among doctors in being offered and accepting red packets, and the attitudes towards such forbidden behavior. This research is the first to identify the gender difference of unethical behavior in medicine. It is based on doctors’ performance and reactions in a real-world working environment, which may reveal the gender differences in unethical behavior more accurately than scenario-based studies.

Firstly, the study found that male doctors were more likely to be offered red packets than female doctors, suggesting that patients and their families are more likely to offer red packets to male doctors. To explore the reasons behind this difference, it is first necessary to determine what the patients’ and their families motivation for sending such red packets. The above mentioned study of 4000 patients from 10 hospitals in China showed that the top three motivations for providing red packets to doctors were: receiving special attention from the doctor, being able to arrange a good doctor for the surgery, and arranging the surgery as soon as possible. Another study involved in-depth interviews with Chinese doctors confirmed this [27]. However, only the senior specialists have the authority to arrange surgery and treatment. Thus, in this research, it seems reasonable to infer that most patients and their families provide red packets to younger doctors out of the hope that the doctor can provide a higher quality of service. From the patients’ perspective, whether a doctor provides high-quality care and services depends on two factors: the doctor’s competence, and the degree of concern the doctor provides. Therefore, patients and their families may be more likely to offer red packets to doctors with a high level of competence. They may be also more likely to offer red packets to doctors whose concern for patients does not meet the patient’s expectation. As Yang makes explicit, "one respondent noted that patients knew of the competence of each doctor and chose to give red packets only to those they believed could solve their acute problems" [37]. Furthermore, "The red packet is paid to ensure that the doctor will commit sufficient time, attention, and perhaps, more importantly, display a good attitude towards the patient" [37].

Thus, there are two possible reasons behind the differences mentioned above. Firstly, compared with female doctors, male doctors’ good attitude and concern for their patients does not meet the expectation of patients and their families. The lack of good attitude and concern motivates patients and their families to offer red packets to male doctors in order to stimulate them to improve their services. Studies have shown that female doctors use more patient-centered communication [38, 39], and provide more psychosocial counselling to their patients than male doctors. This also seems to echo some of the psychological research on the differences between men and women, in which women are more inclined to help others and are more compassionate [1, 40, 41]. Secondly, patients are more likely to believe that male doctors are more competent than female doctors, especially for complex and difficult surgeries. However, studies from the US have shown that female doctors outperform male doctors on key indicators such as mortality within 30 days of surgery, complication rates and post-operative readmission rates [42, 43]. Therefore, the view that male doctors are more competent might simply be a gender bias. Actually, gender bias does exist in medicine. One research revealed that equal number of female and male medical students were tracked for almost 20 years. However, merely 22% of consultants are women within intensive care medicine [44]. Bias against female doctors also exists from the patient’s perspective. One study showed that patients preferred male doctors to do the surgery [45].

There is another possibility that Chinese female doctors indeed underperform their male counterparts on given key aspects. However, on the one hand, it needs future research to confirm, and there has been no report of any such gender difference in this regard in China so far. On the other hand, even if this was to be confirmed by relevant future research, it should not be arbitrarily assumed that male doctors are more competent than female doctors. The reason for this difference may be that female doctors are not given the same opportunities and training as male doctors, due to certain socio-cultural factors. Hence, perhaps what needs to be changed are current medical mechanisms to ensure that female doctors have the same opportunities.

This study also found that among doctors who had been offered red packets, male doctors were more likely to accept red packets than female doctors. As mentioned above, the acceptance of red packets by doctors is not only against the medical ethical code, but also LODC. Therefore, doctors who accepted red packets not only knew that their behavior was ethically and legally prohibited, but also knew that such behavior carried the risk of potential punishment. Even so, male doctors are still more likely to accept red packets than female doctors, confirming previous research that men tend to behave more unethically than women.

There are several possible reasons behind this. Firstly, men are more likely to take risks than women. This has been shown in previous studies [46–48]. In contrast, females may be more influenced by potential sanctions [49]. Secondly, men are more self-confident. Previous studies have established this [50]. And previous studies on red packets indicate an interesting consensus between doctors and patients, that is, accepting red packets is a sign of self-confidence for their competence. Kong and his colleagues mentioned a case in their research which contributes to this view. Accordingly, a patient went to a hospital in Jiangsu (a coastal province in China) for a cardiothoracic surgery and offered a red packet to the doctor. However, the red packet was refused. Then, the patient immediately decided to transfer to another hospital. The doctor was confused. The patient explained, “You are not good enough for this operation because you are not confident enough to accept the red packet.” However, it is not just patients, but also doctors who seem to hold such a view. Yang’s research shows that "taking the red packet is therefore an indicator of the curability of the disease and the doctor's confidence to cure it. If the doctor has confidence, he or she is likely to take the red packet before the operation"[37]. One of the doctors who participated in our questionnaire also stated that “refusing to take the red packet is sometimes perceived by the patient and their families as an indicator that the doctor is not competent in his or her surgery. " Therefore, one possible reason why male doctors are more likely to accept red packets than female doctors is that male doctors are more self-confident than female doctors. Thirdly, women are more prone to experience moral emotions such as guilt and shame than men [2, 5]. Our moral behavior is influenced by the experience of moral emotions and our management of such experiences [5, 6]. A person's proneness to guilt and shame can influence their moral decision making. This may be one of the reasons why female doctors are less likely to accept red packets than male doctors.

Finally, this study found that among doctors who accepted red packets, female doctors were more likely than male doctors to believe that such behavior was not morally wrong, even though they were morally and legally forbidden. In some cases people may obey moral codes for fear of punishment or criticism that they personally do not recognize such codes. Namely, they personally do not find immoral. Our findings show that among doctors who disobeyed the code, female doctors less recognized it than male.

This could suggest, compared with men, women’s behavior is more consistent with the moral codes they recognize, that is, their internal moral principles. Previous research has shown that women are more rule-based and men are more consequence-based [13]. This study seems to confirm that men are more consequence-based, considering that for male doctors who accepted red packets, despite believing that it was morally wrong, the benefits gained by violating such an ethical code greatly outweigh their recognition of the code in deciding whether to accept red packets. However, for the conclusion that women are more rule-based, according to this study, the reason may be that these ethical codes simply coincide with their internal moral principles. This explanation seems to further support the conclusion mentioned above, that women experience moral emotions such as proneness to guilt and shame more strongly than men. After all, experiencing shame suggests that one’s behavior violates an ethical code that one has recognized, rather than just being informed that such behavior is morally forbidden. Future research could focus on the gender differences on the relationship between the violation and the recognition of ethical codes.

These findings not just contribute to the gender difference research on unethical behavior, but also can provide some helpful suggestions to the medical ethics education in China. Male doctors are more likely to accept red packets suggesting that the teaching of medical ethics needs apply some strategies that are more effective for men. Part of doctors who accept red packets actually do not view such behavior as unethical suggesting that medical ethics education needs to pay more attention to help doctors to recognize such ethical codes. Red packets are one of the most critical factors affecting the doctor-patient relationship in China. From the perspective of receiving red packets, perhaps the relationship between male doctors and patients is more complicated than that of female doctors. Therefore an in-depth study of red packets in the medical environment and its gender differences may provide some meaningful theoretical basis for further understanding and analysis of the unsatisfactory doctor-patient relationship in China.

## Limitation

Firstly, the sample size of this study is limited and the snowball sample method may lead to an inevitable potential selection bias consequently. Secondly, even if we have tried to avoid it, there are bound to be some participants who choose not to fill in the real situation because of mistrust. Finally, our participants were drawn from the central 3AAA hospitals in each province and city. Perhaps the situation in primary care units will be different, and this needs further exploration and research in the future.

## Conclusion

Our study has revealed some differences between male doctors and female doctors in their behavior and attitudes towards accepting red packets. Firstly, female doctors were less likely to be offered red packets by patients or their families than male doctors. Secondly, female doctors were less likely to accept red packets which was consistent with the previous studies in which females were generally shown to be more ethical. Thirdly, for doctors who accepted red packets, females were less likely to view such forbidden behavior as morally problematic. This study has important implications for gaining insight into the factors affecting the doctor-patient relationship in the Chinese healthcare environment.

## Supplementary Information

**Additional file 1**. Questionnaire.

**Additional file 2**. Informed consent.

## Data Availability

The datasets used and/or analysed during the current study are available from the corresponding author on reasonable request.
