# Supplementary material for: Gender differences in response to medical red packets (Hongbao, monetary gifts): a questionnaire study on young doctors in China
Source: BMC Med Ethics. 2022 Apr 19;23:44. doi: 10.1186/s12910-022-00781-0 (PMC9019946; doi:10.1186/s12910-022-00781-0)
Supplement: Supplementary file 1 — Additional file 1. Questionnaire. [file 12910_2022_781_MOESM1_ESM.docx]

**Questionnaire**

**1. Your age ( )**

**2. Your sex**

A. Male B. Female

**3. Your educational qualifications**

A. Bachelor's degree B. Master's degree C. Doctorate D. Other

**4. Your current position/title**

A. Postgraduate B. Resident C. Attending physician D. Chief physician

**5. The province in which you are currently working ( )**

**6. Have you ever been offered red packets by patients or their families?**

A. Yes (go to Question 7) B. No (end of the questionnaire)

**7. What did you do when you were first time offered the red packet?**

A. Accepted (go to Question 9)

B. Refused

C. Accepted it and then used it to pay the patient’s medical bill

D. Handed it over to the hospital disciplinary department

E. Other ______ (please write down your reaction)

**8. Have you ever accepted red packets since you started working as a clinician?**

A. No (end of the questionnaire)

B. Yes (go to Question 9)

**9. (This is a multiple-choice question) What are the factors that influence you to accept the red packet?**

A. The complexity of the patient's disease

B. The patient's financial status

C. Whether the patient has medical insurance or not

D. The patient's educational level

E. The patient-physician relationship

**10. Is there a significant change in your attitude towards a patient and in the efficiency and quality of your management of that patient's condition after you have received a red packet from that patient?**

A. Be more patient, but not at the expense of other patients

B. The patient will be given priority over other patients, for example, by being given priority for a bed or surgery

C. No change

**11. What do you think about the acceptance of red packets?**

A. It is unethical to accept extra fees from patients

B. It is Ok to accept red packets. It is the compensation that doctors deserve for their heavy clinical workload

C. It is OK to accept red packets. But the doctors must do their best to treat patients after the acceptance of red packets.

D. Other
