# Supplementary material for: Gender differences in response to medical red packets (Hongbao, monetary gifts): a questionnaire study on young doctors in China
Source: BMC Med Ethics. 2022 Apr 19;23:44. doi: 10.1186/s12910-022-00781-0 (PMC9019946; doi:10.1186/s12910-022-00781-0)
Supplement: Supplementary file 2 — Additional file 2. Informed consent. [file 12910_2022_781_MOESM2_ESM.docx]

**Informed consent**

Hello! We are members of the Medical Humanities Research Team at Nankai University and are conducting a social study about the phenomenon of Chinese doctors receiving red packets. Thank you very much for taking the time to participate in our questionnaire research!

Please read and answer the questions carefully. There is no correct answer to all questions and just choose the option that most accurately expresses your true feelings.

This questionnaire is entirely anonymous and will not violate your privacy. If you have any questions about the content of the survey, please do not hesitate to contact the researcher.

Sincerely
